# Data governance transparency and psychological wellbeing in digital platforms: the roles of trust and emotional regulation

**DOI:** 10.3389/fpsyg.2026.1845779

**Published:** 2026-09-10

**Authors:** Shangqun Wu, Meiqi Yin

**Affiliations:** 1 School of Management, Yangzhou Polytechnic University, Yangzhou, Jiangsu, China; 2 International Business College, Dongbei University of Finance and Economics, Dalian, Liaoning, China

**Keywords:** data governance transparency, digital platforms, emotional regulation, privacy concern, psychological wellbeing, trust

## Abstract

**Introduction:**

Digital platforms increasingly govern users through data collection, inference, sharing, retention, and accountability arrangements, but the psychological consequences of perceived data governance transparency remain underexamined. This study tested whether perceived transparency is associated with platform-related psychological wellbeing through trust and context-specific emotional regulation, and whether privacy concern weakens this relationship.

**Methods:**

Study 1 surveyed 612 adult digital platform users recruited through a professional online panel in mainland China. Confirmatory factor analysis, structural equation modeling, bias-corrected bootstrap mediation with 5,000 resamples, common-method diagnostics, and moderation analysis were conducted. Study 2 used between-subjects scenario experiment with 286 participants assigned to high- or low-transparency descriptions of a fictitious platform. Group comparisons and bootstrap conditional process analyses tested the proposed mechanism.

**Results:**

In Study 1, perceived transparency was positively associated with wellbeing (beta = 0.17, *p* < 0.01), trust (beta = 0.59, *p* < 0.001), and emotional regulation (beta = 0.16, *p* < 0.01). Trust and emotional regulation showed significant separate indirect effects, and the sequential indirect effect was 0.077 (95% CI [0.049, 0.113]). Privacy concern weakened the direct transparency-wellbeing association (interaction beta = −0.08, *p* = 0.018). In Study 2, the high-transparency condition produced higher trust (*d* = 0.84), emotional regulation (*d* = 0.51), and wellbeing (*d* = 0.64); the sequential indirect effect was 0.072 (95% CI [0.036, 0.122]).

**Discussion:**

Perceived data governance transparency may support platform-related psychological wellbeing through an organized process involving trust and emotional regulation, although its benefits are weaker among users with stronger privacy concern. The experimental findings are limited to structured vs. vague policy-text cues and should not be generalized to the full ecology of real-world platform governance.

## 1 Introduction

### 1.1 Digital platforms as psychologically consequential environments

Digital platforms have evolved far beyond their initial role as neutral infrastructures for information exchange or market transactions. They now function as pervasive socio-technical environments that shape how individuals access information, form judgments, regulate attention, interact with others, and interpret everyday experience (Cheong, 2024; Vanden Abeele, 2021; Shin, 2025). In contemporary platform settings, users are not merely exposed to content and services; they are continuously embedded in systems that collect, process, infer, and redistribute data in ways that structure the conditions of participation, visibility, recommendation, and decision making. As a result, digital platforms have become psychologically consequential environments whose influence extends beyond efficiency and convenience to users' cognitive appraisals, emotional responses, and subjective sense of wellbeing (Babu et al., 2025; Chen et al., 2025; Büchi, 2024).

This shift is particularly important because user experience on digital platforms is increasingly mediated by data governance arrangements. Platform rules concerning what data are collected, how those data are interpreted, whether they are shared, and how such processes are communicated to users affect more than institutional legitimacy or legal compliance. These arrangements may also shape whether users perceive the platform environment as understandable, predictable, and controllable (Esmaeilzadeh, 2019; Moon et al., 2025; Schnackenberg and Tomlinson, 2016; Grimmelikhuijsen, 2023). In turn, such perceptions can influence feelings of security, perceived agency, emotional comfort, and broader psychological evaluations of platform use. Put differently, the governance of data is not only an organizational or technical issue; it is also a psychologically meaningful feature of the environment in which users think, feel, and act (Cheong, 2024; Büchi, 2024; Shin, 2025).

From this perspective, data governance should not be treated merely as an external background condition. For platform users, governance-related cues may serve as interpretive signals about whether the platform is trustworthy, whether its operations are aligned with user interests, and whether participation involves manageable or opaque forms of risk. When a platform communicates clearly how personal data are collected, used, protected, and explained, users may experience reduced ambiguity and greater confidence in the interaction context. By contrast, when rules are vague, fragmented, or difficult to interpret, uncertainty may intensify and the platform may be experienced as psychologically taxing (Esmaeilzadeh, 2019; Betzing et al., 2020; Trepte, 2021). Therefore, understanding digital platforms as psychologically consequential environments provides a necessary starting point for examining how perceived data governance conditions may be linked to users' psychological wellbeing.

### 1.2 Why data governance transparency is psychologically relevant but not self-evidently beneficial

Data governance transparency is commonly regarded in policy, governance, and regulatory discussions as a desirable institutional feature. In principle, transparent governance can improve explainability, strengthen accountability, and enhance the predictability of institutional processes (Cheong, 2024; Moon et al., 2025; Schnackenberg and Tomlinson, 2016; Grimmelikhuijsen, 2023). Within digital platforms, transparent communication about data practices may help users better understand how their information is collected, processed, shared, and protected. Such clarity may reduce ambiguity, support informed expectations, and contribute to a more stable sense of control in platform interactions. For these reasons, transparency is often normatively framed as beneficial.

However, in psychological terms, the consequences of data governance transparency are not self-evident. In digital platform contexts, more transparency does not automatically imply more positive user experience. More detailed disclosures may indeed reduce uncertainty and foster trust, but they may also heighten users' awareness of surveillance, privacy vulnerability, data misuse, or the asymmetry of power between platforms and users (Esmaeilzadeh, 2019; Zhu et al., 2025; Lu et al., 2025; Betzing et al., 2020). Information that is intended to reassure may, in some cases, foreground risks that users had previously underestimated. Similarly, disclosures that increase formal transparency may still be experienced as cognitively burdensome, emotionally unsettling, or practically difficult to interpret. Thus, the psychological meaning of transparency depends not only on the amount of information provided, but also on how users subjectively perceive and process that information.

This ambiguity is precisely why the psychological consequences of data governance transparency require theoretical analysis and empirical examination rather than normative assumption. If transparency functions as a cue that reduces uncertainty, enhances predictability, and signals responsibility, it may support trust formation and psychological comfort. If, by contrast, transparency makes risks more salient or underscores users' limited control over data practices, it may amplify unease rather than alleviate it. The key issue, therefore, is not whether transparency is universally good in an abstract governance sense, but whether and through what psychological mechanisms perceived data governance transparency is associated with users' wellbeing in actual platform environments. Framing the question in this way helps avoid a simplistic assumption that transparency must necessarily produce beneficial outcomes, and instead positions transparency as a psychologically meaningful but empirically open condition.

### 1.3 Research gap

Existing research on digital platforms and data governance has generated important insights, but four unresolved issues remain. First, transparency has often been examined as a compliance instrument, a privacy-disclosure feature, or an antecedent of behavioral outcomes such as acceptance, continued use, and platform loyalty (Esmaeilzadeh, 2019; Baruh et al., 2017; Büchi, 2024). These studies clarify why transparent data practices may support institutional legitimacy and user trust, but they pay less attention to whether users experience the platform environment as psychologically comfortable, safe, and emotionally manageable.

Second, the focal construct of transparency is frequently used in a broad manner. In platform research, transparency may refer to readability of information, disclosure of privacy policies, perceived control over data practices, explainability of algorithms, or general openness of organizations. These constructs are related but not identical. Without sharper conceptual boundaries, it is difficult to determine whether observed effects are due to data-governance transparency itself or to adjacent constructs such as information clarity, privacy disclosure, or perceived control. The present study therefore defines perceived data governance transparency as users' perception that the platform's data-governance arrangements are understandable, predictable, and accountable across data collection, use purposes, sharing boundaries, retention, protection, and recourse mechanisms.

Third, the relationship between transparency and trust has often been treated as a relatively direct and terminal association. In such accounts, transparency improves users' evaluation of the platform, and trust then becomes either an outcome or a predictor of adoption-related behavior (Bhattacherjee, 2002; McKnight et al., 2002; Oesterreich et al., 2025). This approach is useful but incomplete for the present research problem. In digital platform environments, trust may also reduce users' need for constant vigilance and may thereby shape how they regulate emotions arising from data-related uncertainty. Thus, the theoretical question is not only whether transparency increases trust, but also how trust participates in a broader psychological process through which governance perceptions become linked to wellbeing.

Fourth, transparency is unlikely to affect all users uniformly. Users differ in their baseline sensitivity to privacy risk and in how they interpret data-governance information. The present manuscript therefore treats privacy concern as a theoretically driven boundary condition rather than as an exploratory subgroup split. This is important because transparent communication may simultaneously reduce ambiguity and make data collection, sharing, and platform discretion more salient. For users with strong privacy concern, the psychological comfort derived from transparency may therefore be weaker than it is for users with lower privacy concern.

Taken together, these gaps indicate the need for a more precise, psychologically grounded account of perceived data governance transparency. The present study moves beyond a simple transparency–trust–outcome logic and examines whether perceived transparency is associated with platform-related psychological wellbeing through a sequential process in which transparency supports trust, trust reduces vigilance and perceived threat, and this more trusting psychological state facilitates context-specific emotional regulation. The study also formally examines privacy concern as a boundary condition that clarifies when and for whom transparency is most likely to yield psychological benefits.

### 1.4 Research objective

In response to these unresolved issues, the present study aims to propose and test an explanatory model of how perceived data governance transparency in digital platforms may be associated with users' psychological wellbeing. Rather than assuming that transparency is inherently beneficial, this study investigates whether transparency-related perceptions are linked to wellbeing and whether such a relationship can be understood through the psychological mechanisms of trust and emotional regulation.

More specifically, this study focuses on perceived data governance transparency as a psychologically salient environmental cue, psychological wellbeing as the focal outcome, and trust and emotional regulation as two central explanatory processes. By doing so, the study seeks to clarify not only whether governance perceptions matter for users' wellbeing, but also how those perceptions may be translated into subjective psychological experience. The purpose is therefore both conceptual and empirical: to develop a theoretically grounded explanatory framework and to examine whether the proposed relationships are supported by evidence from survey and experimental data.

### 1.5 Research questions

To guide the analysis, this study addresses the following research questions:

- RQ1: In digital platform environments, is users' perceived data governance transparency associated with their platform-related psychological wellbeing?
- RQ2: Does trust constitute an important explanatory mechanism linking perceived data governance transparency to platform-related psychological wellbeing?
- RQ3: Does emotional regulation constitute an important explanatory mechanism linking perceived data governance transparency to platform-related psychological wellbeing?
- RQ4: Do trust and emotional regulation form a sequential psychological pathway that jointly explains the relationship between perceived data governance transparency and platform-related psychological wellbeing?
- RQ5: Does privacy concern condition the psychological benefit of perceived data governance transparency?

These questions reflect the central concern of the study: not merely whether transparency matters, but how and for whom a governance-related perception may become psychologically consequential in platform use.

### 1.6 Contributions

This study makes four main contributions. First, it sharpens the construct of perceived data governance transparency in digital platforms. Rather than treating transparency as a synonym for information clarity, privacy-policy disclosure, perceived control, or generalized trust, the study defines it as a user-perceived governance cue concerning the understandability, predictability, and accountability of platform data practices (Schnackenberg and Tomlinson, 2016; Esmaeilzadeh, 2019). This refined definition helps establish construct distinctiveness and reduces conceptual redundancy with adjacent information-systems constructs.

Second, the study extends platform governance research from institutional and behavioral outcomes to platform-related psychological wellbeing. Prior work has widely examined whether transparency promotes trust, acceptance, compliance, or continued use. The present study asks a different question: whether data-governance conditions become part of users' subjective psychological experience within the platform environment. This shifts the outcome focus from what users do to how users feel and psychologically appraise the data-governed platform context.

Third, the study specifies a sequential psychological mechanism linking transparency to wellbeing. The contribution is not merely to add another mediator to the familiar transparency–trust relationship. Rather, the study theorizes trust as an intermediate psychological state that reduces vigilance and perceived vulnerability, thereby supporting users' capacity to regulate unease triggered by platform data practices (Mayer et al., 1995; Gross, 1998; Ning et al., 2024). By placing context-specific emotional regulation after trust, the model explains how governance perceptions may be translated into psychological comfort through both cognitive-relational and affective-regulatory processes.

Fourth, the study introduces privacy concern as a formal boundary condition. This addition prevents the model from assuming that transparency is uniformly beneficial. It specifies that transparency may yield stronger psychological benefits for users who do not strongly interpret data practices through a risk-sensitive lens, while its direct comfort effect may be weaker among users with stronger privacy concern (Malhotra et al., 2004; Baruh et al., 2017; Hsu et al., 2022). In this way, the study contributes a boundary-aware account of transparency, trust, emotional regulation, and platform-related psychological wellbeing.

## 2 Literature review and hypothesis development

### 2.1 Perceived data governance transparency in digital platforms

Data governance transparency in digital platforms refers to the extent to which platform rules and practices concerning data collection, use, sharing, retention, protection, user rights, and accountability are made visible and understandable to users (Schnackenberg and Tomlinson, 2016; Esmaeilzadeh, 2019; Grimmelikhuijsen, 2023). The present study focuses on *perceived* data governance transparency rather than the objective existence of formal disclosure documents. Perceived data governance transparency is defined here as users' subjective judgment that a platform's data-governance arrangement is communicated in a clear, comprehensible, predictable, and accountable manner.

This definition contains three boundary conditions. First, the construct is *governance-specific*: it concerns data collection, purpose specification, sharing boundaries, retention, protection, accountability, and user recourse, rather than all forms of platform openness. Second, it is *perception-based*: users may perceive a platform as opaque even when legal documents exist, if the governance arrangement is difficult to understand or fragmented across interfaces. Third, it is *not equivalent to control*: users may understand data practices without being able to change them, and they may have nominal controls without understanding how the governance system works.

This distinction is important because users rarely encounter data governance as a complete institutional system. Instead, they infer governance from privacy notices, consent interfaces, in-app explanations, account settings, user-rights statements, support channels, notification practices, and accumulated platform experience. A platform may provide legally detailed documentation while still being psychologically opaque to users. Conversely, a concise and structured explanation may create stronger perceived transparency if users can understand what data are collected, why they are used, whether they are shared, how long they are retained, how they are protected, and what rights or remedies are available (Betzing et al., 2020; Moon et al., 2025).

To address potential conceptual overlap, perceived data governance transparency is distinguished from several adjacent constructs. It is broader than information clarity because it concerns the perceived understandability of the platform's data-governance arrangement, not only the readability of a specific message. It is broader than privacy disclosure because it includes not only disclosure of privacy practices, but also accountability, predictability, protection, retention, and responsibility boundaries. It differs from perceived control because transparency concerns whether users understand how data governance works, whereas control concerns whether users believe they can act upon or alter those practices. It differs from algorithmic transparency because it focuses on data-governance arrangements rather than the technical logic of automated decisions or recommendation algorithms. Finally, it differs from trust because transparency is theorized as an antecedent governance cue, whereas trust is a downstream relational evaluation of the platform's reliability, honesty, and responsibility (Mayer et al., 1995; McKnight et al., 2002).

From this perspective, perceived data governance transparency is a psychologically salient environmental cue. It may reduce ambiguity and help users interpret the platform environment as more predictable and accountable. At the same time, transparency should not be assumed to be uniformly beneficial. Clearer information may also make data-related risks more salient, especially for users with strong privacy concerns (Baruh et al., 2017; Malhotra et al., 2004; Zhu et al., 2025). Therefore, the present study treats perceived data governance transparency as an empirically open governance cue whose psychological consequences depend on users' trust formation, emotional regulation, and privacy-risk sensitivity. Table 1 summarizes these conceptual boundaries.

**Table 1:** Conceptual boundaries of perceived data governance transparency.

Adjacent construct	Main focus	Difference from perceived data governance transparency
Information clarity	Whether a message is easy to read and understand	Transparency concerns the perceived governance arrangement, not only the clarity of a single message or document.
Privacy disclosure	Whether privacy-related information is disclosed	Transparency includes disclosure, but also covers use purposes, sharing boundaries, retention, protection, accountability, and user recourse.
Perceived control	Whether users feel able to influence or manage data practices	Transparency concerns whether users can understand governance rules; control concerns whether users can act on them.
Algorithmic transparency	Whether automated decisions or recommendation logic are explainable	Data governance transparency concerns the rules and responsibilities governing data, not only algorithmic decision logic.
Trust	Whether users view the platform as reliable, honest, and responsible	Transparency is an antecedent governance cue; trust is a downstream psychological evaluation.

### 2.2 Psychological wellbeing in platform use contexts

Platform-related psychological wellbeing refers to users' positive subjective psychological experience within a digital platform environment. In this study, it captures users' sense of psychological comfort, safety, ease, emotional manageability, and non-threatening platform experience when interacting with a data-governed platform. This construct is intentionally context-specific. It does not refer to global life satisfaction or general psychological wellbeing across all life domains, but to users' psychological state when using and interpreting a platform whose services depend on data collection, processing, inference, sharing, and governance (Ryff, 1989; Vanden Abeele, 2021; Büchi, 2024).

This contextualization is necessary because digital platform use involves forms of uncertainty that are not fully captured by general wellbeing measures. Users interact with algorithmic recommendations, invisible data flows, personalized services, privacy notices, data-sharing arrangements, and shifting boundaries of visibility and control. Their psychological wellbeing in this context may therefore depend on whether the platform environment feels intelligible, responsible, predictable, safe, and emotionally manageable (Cheong, 2024; Smits et al., 2022; Shin, 2025).

Platform-related psychological wellbeing is also distinct from several traditional information-systems constructs. It is not the same as user satisfaction, which mainly reflects an evaluative judgment about whether a service meets expectations. It is not the same as system affect, which usually captures immediate positive or negative feelings toward a technology. It is also not equivalent to perceived usefulness or continuance intention, which focus on instrumental value and future behavior. The construct used in this study is broader and more governance-sensitive: it concerns whether users feel psychologically comfortable, safe, and mentally at ease within a platform environment where their data are continuously collected, processed, inferred, shared, and governed. Table 2 summarizes the boundaries between platform-related psychological wellbeing and adjacent constructs.

**Table 2:** Boundaries of platform-related psychological wellbeing.

Adjacent construct	Main focus	Difference from platform-related psychological wellbeing
General life wellbeing	Overall evaluation of life quality and functioning	The present construct is limited to psychological experience within platform use contexts.
User satisfaction	Whether a system or service meets expectations	Platform-related wellbeing emphasizes comfort, safety, ease, and emotional manageability, not only evaluative satisfaction.
System affect	Immediate positive or negative feelings toward a system	Platform-related wellbeing is more stable and context-specific, focusing on the perceived psychological quality of the data-governed environment.
Perceived usefulness	Instrumental value of a system for task performance	The present construct concerns subjective psychological experience rather than functional utility.
Continuance intention	Intention to keep using a platform	Platform-related wellbeing is an experiential outcome, not a behavioral intention.

Accordingly, platform-related psychological wellbeing provides a suitable outcome variable for examining data governance transparency. If users perceive platform data practices as opaque, unpredictable, or institutionally distant, they may experience the platform as psychologically burdensome even when it remains functionally useful. Conversely, when users perceive data governance as understandable and responsibly communicated, they may experience greater psychological comfort and emotional ease. This study therefore examines psychological wellbeing as a platform-specific subjective state shaped by governance-related perceptions rather than as a generic indicator of happiness, service satisfaction, or system affect.

### 2.3 Theoretical foundations

#### 2.3.1 Psychological economics perspective

Psychological economics highlights that individuals do not respond to uncertain environments solely through objective calculation. Rather, they interpret uncertainty through subjective judgments of risk, control, anticipated loss, and psychological cost (Kahneman and Tversky, 1979; Thaler, 1980). When individuals face information asymmetry or unclear institutional arrangements, they form internal evaluations about whether the environment is manageable, whether hidden risks may exist, and how much cognitive and emotional effort is required to navigate the situation. Such evaluations can affect not only decisions and behaviors, but also subjective welfare and emotional experience (Kahneman and Tversky, 1979; Thaler, 1980).

In digital platforms, data governance conditions can be viewed as part of the institutional environment through which users interpret uncertainty. When data practices are communicated in ways users perceive as clear and understandable, users may experience the environment as more interpretable and predictable. This does not necessarily mean that risk is objectively reduced. Instead, perceived transparency may reshape users' subjective judgments about how much uncertainty they face and how much control they have over the situation. From a psychological economics perspective, the significance of transparency lies in its potential to alter perceived risk exposure and psychological transaction costs associated with platform participation.

This perspective is particularly useful because it avoids assuming that transparency has uniformly positive effects. More information may reassure users in some cases, but it may also reveal risks, complexity, or power asymmetries that increase discomfort. Thus, transparency is theoretically relevant not because it is necessarily beneficial, but because it may change the terms on which users psychologically evaluate the platform environment. If such changes affect perceived uncertainty and control, they may also shape broader psychological outcomes, including wellbeing.

In the present study, psychological economics is used primarily as an interpretive perspective rather than as a directly measured component of the empirical model. It provides the broader conceptual rationale for why governance-related cues may matter psychologically, while the specific mechanisms tested in the model are trust and emotional regulation.

#### 2.3.2 Trust theory

Trust theory suggests that trust is more likely to emerge when institutional arrangements are perceived as understandable, predictable, and consistent with accepted expectations of responsibility (Mayer et al., 1995; Schnackenberg and Tomlinson, 2016). Trust reduces the need for constant vigilance and allows individuals to engage with complex systems without continuously monitoring every potential risk. In environments characterized by information asymmetry or limited user control, trust becomes especially important because it serves as a psychological bridge between incomplete knowledge and continued participation (Mayer et al., 1995; Oesterreich et al., 2025; Grimmelikhuijsen, 2023).

Digital platforms are prototypical contexts in which such trust dynamics matter. Users rarely possess full knowledge of how their data are processed, inferred, or shared. As a result, they rely not only on explicit policies but also on broader cues about whether the platform appears reliable, honest, and accountable. Perceived data governance transparency may contribute to these judgments by signaling that platform practices are not entirely hidden, arbitrary, or unstructured (Esmaeilzadeh, 2019; Ning et al., 2024; Betzing et al., 2020; Grimmelikhuijsen, 2023). When a platform's data-related rules are experienced as clearer and more predictable, users may become more willing to view the platform as trustworthy (Esmaeilzadeh, 2019; McKnight et al., 2002; Oesterreich et al., 2025).

Importantly, trust is not only relevant to behavioral acceptance or continued usage; it is also psychologically consequential in itself. Trust may reduce anxiety, lower perceived vulnerability, and support a more secure and positive experience of platform engagement (Mayer et al., 1995; McKnight et al., 2002). For this reason, trust is theoretically positioned in this study not merely as a final outcome of transparency, but as an explanatory mechanism through which governance perceptions may become relevant to psychological wellbeing.

#### 2.3.3 Emotion regulation theory

Emotion regulation theory emphasizes that individuals actively manage their emotional responses when confronting uncertainty, ambiguity, or potentially stressful situations (Gross, 1998; Gross and John, 2003). Such regulation may involve reappraising a situation, redirecting attention, reducing emotional reactivity, or maintaining psychological balance when external conditions are difficult to interpret.

In digital platform environments, emotional regulation has a specific governance-related meaning. Users are often exposed to latent concerns about privacy loss, surveillance, algorithmic inference, data misuse, and platform power asymmetry. These concerns do not always arise from direct harm; they may emerge from uncertainty about what the platform knows, how data are combined, whether information is shared, and whether users have meaningful recourse (Malhotra et al., 2004; Trepte, 2021; Zhu et al., 2025). Under these conditions, emotional regulation refers to users' perceived ability to manage unease, suspicion, discomfort, or anxiety triggered by platform data-governance cues.

The present study therefore conceptualizes emotional regulation as context-specific rather than trait-general. The focus is not on whether users are generally good at regulating emotions in everyday life. Instead, the focus is on whether users can maintain emotional stability when interpreting platform data practices and data-related uncertainty. This distinction is central to the theoretical contribution of the study. It links emotion regulation theory to platform governance by showing how governance cues may shape not only cognitive evaluations such as trust, but also users' emotional capacity to experience the platform environment as manageable.

Perceived data governance transparency may facilitate this process when it makes data practices more understandable and less arbitrary. If users can interpret what data are collected, why they are used, how they are protected, and what responsibility boundaries exist, they may find it easier to regulate data-related unease. By contrast, opaque or vague governance communication may intensify emotional demands because users must manage uncertainty without a clear basis for interpretation. Thus, emotional regulation is theorized as a mechanism through which governance perceptions become linked to platform-related psychological wellbeing.

### 2.4 Hypothesis development

#### 2.4.1 Data governance transparency and psychological wellbeing

The preceding discussion suggests that perceived data governance transparency may be psychologically consequential because it can shape how users interpret ambiguity, predictability, and control in the platform environment. When users perceive data governance practices as clearer and more understandable, they may experience less environmental ambiguity and a stronger sense that the platform context is interpretable. Such perceptions may support emotional comfort, reduce diffuse uncertainty, and contribute to a more positive subjective experience of platform use (Esmaeilzadeh, 2019; Cheong, 2024; Moon et al., 2025; Betzing et al., 2020). At the same time, as noted earlier, transparency is not assumed to be universally beneficial in all circumstances. Its relevance lies in its capacity to structure users' interpretations of the environment in ways that may matter for wellbeing.

Given this reasoning, the present study expects that higher perceived data governance transparency is more likely to be associated with higher psychological wellbeing in digital platform use contexts. This expectation is framed as an empirical proposition rather than a self-evident conclusion, and it serves as the starting point for the mechanism-based analysis developed below.

**H1:** *Perceived data governance transparency is positively associated with psychological wellbeing among digital platform users*.

#### 2.4.2 Mediating role of trust

Trust theory suggests that transparency may matter not only because it directly affects subjective comfort, but also because it influences whether users perceive the platform as reliable, honest, and responsible (Mayer et al., 1995; Esmaeilzadeh, 2019; Ning et al., 2024; Schnackenberg and Tomlinson, 2016). If data governance arrangements are experienced as clearer and more predictable, users may feel that the platform is less arbitrary and less likely to exploit hidden asymmetries. In this sense, perceived transparency may support the formation of trust by reducing interpretive uncertainty around institutional intentions and practices (Esmaeilzadeh, 2019; McKnight et al., 2002; Oesterreich et al., 2025; Grimmelikhuijsen, 2023).

Higher trust, in turn, may be associated with better psychological wellbeing. When users trust a platform, they may feel less vulnerable, less compelled to remain vigilant, and more psychologically secure in the interaction environment (Mayer et al., 1995; Ryff, 1989). Such effects can contribute to a more positive and stable experience of platform use. Accordingly, trust may function as an important mechanism through which governance-related perceptions become relevant to users' psychological wellbeing.

Based on this logic, the present study proposes that trust mediates the relationship between perceived data governance transparency and psychological wellbeing.

**H2:** *Trust mediates the relationship between perceived data governance transparency and psychological wellbeing*.

#### 2.4.3 Mediating role of emotional regulation

Perceived transparency may also matter because it shapes the emotional demands users face in platform environments. When governance cues make the environment feel clearer and more interpretable, users may be better able to maintain emotional stability in the face of data-related uncertainty. By contrast, when users perceive platform data practices as opaque or difficult to understand, unease and suspicion may become more difficult to manage (Gross, 1998; Gross and John, 2003; Chen et al., 2025; Vanden Abeele, 2021). In this way, perceived transparency may be associated with how effectively users regulate emotions arising from their interaction with the platform.

Emotion regulation is relevant to psychological wellbeing because individuals who are better able to manage anxiety, discomfort, or uncertainty are more likely to maintain a positive subjective state (Gross and John, 2003; Ryff, 1989; Chen et al., 2025). In digital contexts, this may translate into greater emotional comfort, reduced strain, and a more manageable experience of platform use. Therefore, emotional regulation may represent a second explanatory pathway connecting perceived data governance transparency and psychological wellbeing.

Accordingly, the present study proposes the following hypothesis:

**H3:** *Emotional regulation mediates the relationship between perceived data governance transparency and psychological wellbeing*.

#### 2.4.4 Sequential mechanism of trust and emotional regulation

The two mechanisms discussed above may not operate independently. Trust may itself help shape emotional regulation by reducing vigilance, perceived threat, and anticipatory unease. When users trust a platform, they may be less likely to experience data governance conditions as psychologically threatening, and therefore more able to maintain emotional balance. From this perspective, trust may precede emotional regulation in a sequential process through which governance perceptions are translated into wellbeing (Mayer et al., 1995; Gross, 1998; Ning et al., 2024).

Under this logic, perceived data governance transparency may first foster trust by making the platform appear more understandable and accountable. Greater trust may then facilitate more stable emotional regulation in response to data-related uncertainty. In turn, more effective emotional regulation may be associated with better psychological wellbeing. This sequential mechanism has not been sufficiently examined in prior research on digital platform governance, and thus warrants empirical testing.

The present study therefore proposes a chain mediation hypothesis:

**H4:** *Trust and emotional regulation sequentially mediate the relationship between perceived data governance transparency and psychological wellbeing*.

#### 2.4.5 Moderating role of privacy concern

Although perceived data governance transparency is expected to support trust and wellbeing on average, its psychological effect should not be assumed to be uniform across all users. Subjective responses to governance information depend on how users appraise data-related risk. Privacy concern represents a theoretically important boundary condition because users with stronger privacy concern are more attentive to data collection and sharing, more sensitive to potential misuse, and more likely to interpret governance information through a risk-focused lens (Malhotra et al., 2004; Baruh et al., 2017; Hsu et al., 2022).

For these users, transparency may have two competing implications. On the one hand, clear governance communication can reduce ambiguity and provide a basis for trust. On the other hand, the same communication can make the scope of data collection, sharing, retention, and platform discretion more salient. As a result, transparency may not always translate into psychological comfort. Users with stronger privacy concern may understand platform data practices more clearly, yet still feel uneasy because the transparency cue reminds them of the breadth and complexity of data governance. This logic is consistent with the view that transparency is psychologically consequential but not automatically reassuring.

Privacy concern is therefore incorporated into the structural model as a formal moderating variable rather than as a *post-hoc* median-split robustness check. Among possible user-level moderators, privacy concern is selected because it is theoretically proximal to the focal governance domain and directly captures baseline sensitivity to platform data risk. Algorithmic literacy may also shape responses to transparency, especially in algorithm-intensive platforms, but it was not the focal measured boundary condition in the present design. The current model therefore tests privacy concern as the primary moderator and discusses algorithmic literacy as a promising extension for future research.

Accordingly, the present study expects that the positive association between perceived data governance transparency and platform-related psychological wellbeing will be weaker among users with stronger privacy concern. Users with lower privacy concern may interpret transparent data governance mainly as a signal of responsibility, predictability, and user-centered communication. Users with higher privacy concern may still value transparency, but may also process the same information as evidence of complex data dependence and possible exposure.

**H5:** *Privacy concern moderates the relationship between perceived data governance transparency and platform-related psychological wellbeing, such that the positive association is weaker among users with higher privacy concern*.

### 2.5 Conceptual framework

**Figure 1:**
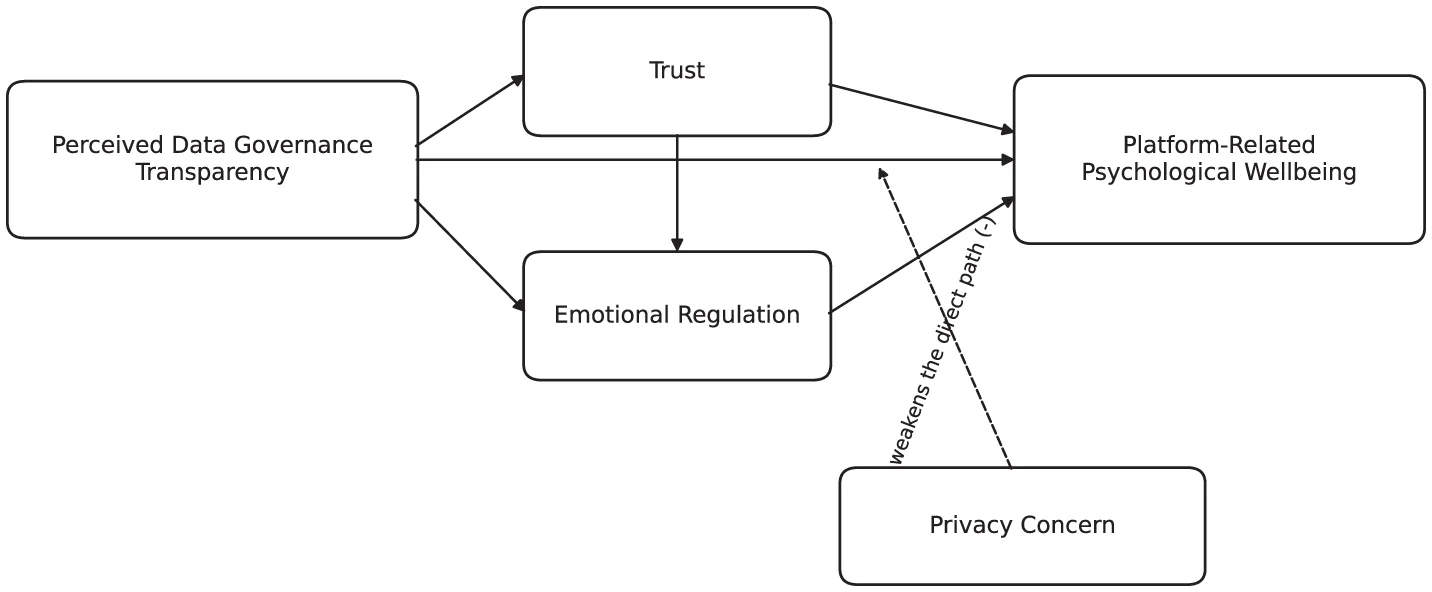
Proposed conceptual framework. The model links perceived data governance transparency to platform-related psychological wellbeing through trust and context-specific emotional regulation. Privacy concern is included as a boundary condition that weakens the direct transparency–wellbeing association among users who are more sensitive to data-related risks.

Based on the foregoing arguments, this study develops a conceptual framework in which perceived data governance transparency is linked to platform-related psychological wellbeing both directly and indirectly through trust and emotional regulation. The model further specifies a sequential pathway in which perceived transparency supports trust, trust facilitates context-specific emotional regulation, and emotional regulation is associated with higher platform-related psychological wellbeing. In addition, privacy concern is incorporated as a boundary condition that may weaken the direct psychological benefit of perceived transparency for users who are especially sensitive to data-related risks. Importantly, the framework does not assume that transparency is universally beneficial. Rather, it treats perceived transparency as a governance-related cue whose psychological consequences depend on the mechanisms and boundary conditions through which users interpret the platform environment. Figure 1 presents the proposed conceptual framework. The following sections describe the two-study design used to examine the direct association, the mediation mechanisms, the sequential pathway, and the moderating role of privacy concern.

## 3 Methodology

### 3.1 Overall research design

To examine the proposed explanatory model, this study adopts a two-study design that combines a survey study with a scenario experiment. The purpose of this design is not to assume that the proposed relationships must hold, but to evaluate whether the expected pattern is supported across complementary forms of evidence. Study 1 uses a cross-sectional survey to examine whether perceived data governance transparency is associated with psychological wellbeing among digital platform users and whether trust and emotional regulation help explain this association. This study is intended to assess the correlational structure of the proposed model in a relatively broad user sample. Study 2 uses a scenario-based experiment to manipulate the level of data governance transparency and to examine whether differences in transparency conditions are associated with systematic differences in trust, emotional regulation, and psychological wellbeing. By complementing the survey evidence with an experimental design, the study aims to strengthen the interpretive basis for the proposed mechanism pattern.

### 3.2 Study 1: survey test of the transparency-wellbeing mechanism

#### 3.2.1 Participants and data collection

Study 1 was designed to capture users' subjective perceptions of data governance transparency and their psychological experience in actual platform use contexts. The target population consisted of adult users with recent and repeated experience using digital platforms, including social media platforms, e-commerce platforms, mobility platforms, and life-service platforms. A summary of the Study 1 sample is provided in Table 3.

**Table 3:** Profile of the Study 1 sample (*N* = 612).

Category	Item	Value
Data collection period	Survey fieldwork	February 1, 2025 to February 2, 2026
Recruitment source	Sample source	Professional online panel, mainland China
Eligibility criteria	Age	≥18 years
Recent use	Platform experience	Used at least one digital platform in past 3 months
Usage frequency	Minimum platform use	At least 3 days per week
Daily duration	Minimum daily use	At least 30 min per day
Initial sample	Returned questionnaires	742
Final sample	Valid questionnaires	612
Effective rate	Valid/initial	82.48%
Gender	Female	52.29%
Gender	Male	47.71%
Age	Mean (SD)	31.84 (8.27)
Education	Bachelor's degree or above	68.14%
Platform type	Social platforms	34.97%
Platform type	E-commerce platforms	29.58%
Platform type	Mobility platforms	16.34%
Platform type	Life-service platforms	19.12%
Usage intensity	Days per week, mean (SD)	6.02 (1.21)
Usage intensity	Hours per day, mean (SD)	2.87 (1.64)

Data were collected through a professional online survey panel in mainland China over a one-year period from February 1, 2025 to February 2, 2026. The extended collection window was used to improve sample heterogeneity and reduce the likelihood that responses would be unduly influenced by a narrowly concentrated temporal context. Before entering the main questionnaire, respondents completed screening questions. Participants were eligible only if they were at least 18 years old, had used at least one digital platform within the past three months, used digital platforms at least three days per week on average, and reported an average daily use duration of at least 30 minutes. Respondents who did not meet these criteria were automatically screened out by the survey system.

Several data-quality procedures were applied. Responses were excluded if the questionnaire was incomplete, if the respondent failed attention-check items, if the completion time was shorter than one-third of the median completion time, or if evident straightlining appeared across the core scale items. A total of 742 responses were initially obtained. After data cleaning, 612 valid responses were retained for analysis, yielding an effective response rate of 82.48%.

The adequacy of the sample size was assessed before model estimation. A priori power analysis indicated that a sample of at least 545 respondents would be required to detect a small-to-moderate effect size (*f*^2^ = 0.03) with nine predictors, α = 0.05, and statistical power above 0.80. The retained sample of 612 therefore exceeded this requirement. This sample size was also adequate for confirmatory factor analysis, structural equation modeling, bootstrap mediation analysis, and supplementary subgroup tests (Hair et al., 2021; Kline, 2023).

The final sample covered a broad range of platform use experiences. Among valid respondents, 34.97% primarily used social platforms, 29.58% primarily used e-commerce platforms, 16.34% primarily used mobility platforms, and 19.12% primarily used life-service platforms. The mean age of respondents was 31.84 years (SD = 8.27), 52.29% were female, and 68.14% reported a bachelor's degree or above. On average, respondents reported using digital platforms 6.02 days per week (SD = 1.21) and 2.87 h per day (SD = 1.64).

#### 3.2.2 Measures

All focal constructs were measured using multi-item scales grounded in established psychometric logic and adapted to the digital platform context of the present study. The purpose of these measures was to capture users' subjective perceptions and psychological experiences rather than objective institutional characteristics. Unless otherwise noted, all focal items were rated on a 7-point Likert scale ranging from 1 (strongly disagree) to 7 (strongly agree). To improve replicability, the full measurement items are reported in Supplementary Appendix B.

##### 3.2.2.1 Perceived data governance transparency

Perceived data governance transparency was measured with a 6-item scale developed with reference to transparency and information-clarity research and reworded for digital platform use contexts (Schnackenberg and Tomlinson, 2016; Esmaeilzadeh, 2019; Betzing et al., 2020). The items assessed whether respondents perceived the focal platform's data collection rules, purpose statements, sharing boundaries, protection mechanisms, retention-related explanations, and responsibility boundaries as clear, understandable, and predictable. This operationalization was designed to capture users' perceived understanding of the platform's data-governance arrangement. It was not intended to measure the objective amount of legal policy text, general information clarity, or perceived behavioral control.

##### 3.2.2.2 Trust

Trust was measured using a 5-item platform trust scale adapted from prior research on institutional trust and digital service trust (Mayer et al., 1995; McKnight et al., 2002; Bhattacherjee, 2002). The items assessed the extent to which users perceived the focal platform as reliable, honest, responsible, dependable, and unlikely to misuse user interests.

##### 3.2.2.3 Emotional regulation

Emotional regulation was measured using five items adapted from existing emotion-regulation scales, with wording adjusted to platform-related uncertainty and data-governance situations (Gross, 1998; Gross and John, 2003). The items focused on respondents' perceived ability to manage unease, concern, or discomfort when facing platform data-related information. In this study, emotional regulation is operationalized as a context-specific construct rather than as a broad trait-level disposition. More specifically, it refers to users' perceived capacity to maintain emotional stability and regulate negative reactions in response to platform data-governance cues and data-related uncertainty.

##### 3.2.2.4 Platform-related psychological wellbeing

Platform-related psychological wellbeing was measured with six items adapted from prior wellbeing and psychological-comfort measures (Ryff, 1989; Vanden Abeele, 2021; Vera Cruz et al., 2025). The scale focused on respondents' positive subjective experience in using digital platforms, including feelings of psychological comfort, safety, ease, emotional manageability, and overall positive mental experience. The construct is operationalized as platform-related psychological wellbeing rather than as general life wellbeing, user satisfaction, system affect, perceived usefulness, or continuance intention.

##### 3.2.2.5 Privacy concern

Privacy concern was measured with a 4-item scale because users with stronger baseline privacy concerns may interpret governance cues differently from other users (Malhotra et al., 2004; Baruh et al., 2017; Hsu et al., 2022). Privacy concern was included as a covariate in the baseline structural model and was then formally tested as the moderator specified in H5. This design treats privacy concern as a theoretically motivated boundary condition rather than as an exploratory subgroup variable.

##### 3.2.2.6 Marker variable for common method assessment

To supplement the latent method factor analysis, a theoretically unrelated marker variable was included for common method diagnostics. The marker items concerned general preference for simple interface appearance and were not conceptually tied to data governance transparency, trust, emotional regulation, or platform-related psychological wellbeing. The marker-variable specification was used only to assess whether the substantive relationships remained stable after accounting for potential shared method variance.

##### 3.2.2.7 Control variables

To reduce the possibility that the observed relationships were driven by demographic or usage differences, several control variables were included: age, gender, education level, platform use frequency, daily usage time, and primary platform type. Table 4 provides a concise overview of the Study 1 measures.

**Table 4:** Measures used in Study 1.

Construct	Items	Scale format	Content focus
Perceived data governance transparency	6	7-point Likert	Understandability, predictability, and accountability of data-governance practices
Trust	5	7-point Likert	Reliability, honesty, responsibility, and dependability
Emotional regulation	5	7-point Likert	Regulation of unease and emotional stability in data-related contexts
Platform-related psychological wellbeing	6	7-point Likert	Psychological comfort, safety, ease, and positive platform experience
Privacy concern	4	7-point Likert	Baseline concern about privacy and data misuse; moderator in H5
Marker variable	3	7-point Likert	Theoretically unrelated interface-preference marker for CMV diagnostics
Demographic and usage controls	Six variables	Mixed format	Age, gender, education, usage frequency, daily use time, and platform type

#### 3.2.3 Reliability and validity assessment

To evaluate the psychometric adequacy of the measurement model, several reliability and validity checks were planned. Internal consistency reliability was assessed using Cronbach's alpha and composite reliability (CR), with values above 0.70 considered acceptable. Convergent validity was assessed using standardized factor loadings and average variance extracted (AVE), with standardized loadings preferably above 0.60 and AVE values above 0.50 (Fornell and Larcker, 1981; Hair et al., 2021; Kline, 2023). Discriminant validity was examined using two complementary approaches. First, the square root of AVE for each construct was compared against inter-construct correlations following the Fornell–Larcker criterion (Fornell and Larcker, 1981). Second, the heterotrait–monotrait ratio (HTMT) was examined to ensure that conceptually distinct constructs remained empirically distinguishable (Henseler et al., 2015). Confirmatory factor analysis (CFA) was then used to compare the hypothesized four-factor measurement model with alternative nested models, thereby evaluating whether the focal constructs exhibited acceptable structural separation (Brown, 2015; Kline, 2023). In line with common psychometric practice, acceptable internal consistency was indicated by Cronbach's alpha and composite reliability values above 0.70, convergent validity was supported by AVE values above 0.50 and satisfactory factor loadings, and discriminant validity was evaluated using the Fornell–Larcker criterion and HTMT values (Fornell and Larcker, 1981; Henseler et al., 2015; Hair et al., 2021).

#### 3.2.4 Common method considerations

Because the main variables in Study 1 were collected from the same respondents, common method variance was treated as both a design issue and an empirical assessment issue. Procedurally, the survey was designed to reduce method-driven inflation in several ways. First, anonymity was emphasized to reduce evaluation apprehension. Second, the order of scale blocks was arranged to reduce direct cueing between predictor and outcome variables. Third, scale wording was kept concise and context-specific to minimize ambiguity. Fourth, attention checks and speed-screening criteria were used to reduce low-quality responses (Podsakoff et al., 2003).

Statistically, Harman's single-factor test was used only as an initial diagnostic and was not treated as sufficient evidence by itself. A single-factor confirmatory factor analysis model was compared with the hypothesized multi-factor measurement model to assess whether the item covariance could be reduced to one general factor (Brown, 2015; Kline, 2023). In addition, a latent method factor model was estimated as the primary robustness diagnostic. In this model, items were allowed to load on their theoretical constructs and also on a common latent method factor. The substantive structural paths were then compared with those in the baseline model to evaluate whether the main conclusions were stable after accounting for shared method variance.

A marker-variable specification was also estimated as a supplementary diagnostic. The marker variable was intentionally unrelated to the theoretical model and was used to partial out method-related covariance that could arise from common response format, common source, or general response tendency. By combining procedural remedies, single-factor CFA comparison, latent method factor analysis, and marker-variable analysis, the study provides a more rigorous defense against common method bias than reliance on Harman's single-factor test alone. These analyses were used to assess whether the proposed sequential mediation pattern was primarily attributable to method-driven inflation.

#### 3.2.5 Analytical strategy

The analysis of Study 1 proceeded in seven steps. First, descriptive statistics and bivariate correlations were computed for all focal variables to examine initial association patterns. Second, confirmatory factor analysis was conducted to assess the measurement model and to evaluate convergent validity, discriminant validity, and model fit. Third, the hypothesized structural equation model was estimated to test the direct associations among perceived data governance transparency, trust, emotional regulation, and platform-related psychological wellbeing while controlling for demographic and usage variables. Fourth, bias-corrected bootstrap mediation analysis with 5,000 resamples was used to test the indirect effects through trust, emotional regulation, and the sequential pathway from trust to emotional regulation (Hayes, 2022). Fifth, common method variance and multicollinearity diagnostics were conducted, including single-factor CFA comparison, latent method factor analysis, marker-variable analysis, and variance inflation factor assessment. Sixth, privacy concern was formally tested as the moderating variable specified in H5 by estimating the interaction between perceived data governance transparency and privacy concern after mean-centering the relevant variables. Seventh, supplementary analyses were conducted to assess the stability of the model across major platform-use groups.

For transparency of reporting, Table 5 summarizes the main analyses conducted in the two studies.

**Table 5:** Analytical overview across the two studies.

Study	Analytical stage	Method	Purpose
Study 1	Preliminary analysis	Descriptive statistics and correlations	Examine variable distributions and initial associations
Study 1	Measurement assessment	CFA	Assess construct validity and factor structure
Study 1	Structural testing	SEM	Evaluate the proposed explanatory model
Study 1	Mechanism testing	Bootstrap mediation, 5,000 resamples	Test indirect and sequential mediation effects
Study 1	Method-bias diagnostics	Single-factor CFA, latent method factor, marker-variable analysis	Assess common method variance
Study 1	Collinearity diagnostics	Variance inflation factors	Assess multicollinearity among predictors
Study 1	Boundary-condition test	Moderation analysis	Examine privacy concern as a moderator
Study 2	Manipulation assessment	Independent-samples *t*-test	Verify transparency manipulation
Study 2	Condition comparison	*t*-test/ANOVA	Examine group differences in psychological responses
Study 2	Mechanism testing	Conditional process analysis with bootstrap	Test indirect and sequential mechanism under manipulation

### 3.3 Study 2: scenario-based experimental validation

#### 3.3.1 Experimental rationale

The survey study was designed to examine whether the variables in the proposed model exhibited the expected pattern of association in users' reported experiences. However, survey evidence alone provides limited leverage for interpreting directionality. To complement the correlational findings, Study 2 was designed as a scenario experiment in which perceived data governance transparency was manipulated through controlled platform descriptions. The purpose of this experiment was to assess whether different transparency conditions were associated with systematic differences in trust, emotional regulation, and psychological wellbeing.

#### 3.3.2 Experimental design

Study 2 adopted a single-factor between-subjects design with two conditions: high data governance transparency and low data governance transparency. To avoid contamination from pre-existing platform attitudes, the experiment used a fictitious integrated digital platform named *LinkLife*. The platform was described as a commonly used digital service platform that combined social communication, e-commerce, mobility booking, and local life services. This design allowed participants to imagine a plausible platform environment without bringing strong prior brand evaluations into the task.

The two experimental conditions differed in the presentation of data-governance information. In the high-transparency condition, the platform's data practices were described using concise, well-structured, and user-oriented explanations, including what data were collected, why they were collected, how long they were retained, whether they were shared with third parties, what user rights applied, and where users could seek support. In the low-transparency condition, the same general topic was described in vague, fragmented, and minimally interpretable terms, with limited explanation of purposes, unclear boundaries of sharing, and little practical guidance about user rights or recourse.

The scope of this manipulation is intentionally bounded. The experiment manipulated a policy-text-based transparency cue, not the full ecology of real-world platform governance. Real platforms communicate transparency through multiple channels, including privacy notices, interface design, consent flows, privacy dashboards, notification systems, algorithmic explanations, and user-rights mechanisms (Betzing et al., 2020; Paunov et al., 2022). Therefore, Study 2 should be interpreted as a controlled test of whether structured vs. vague data-governance descriptions influence users' psychological responses. It should not be interpreted as evidence that a static text block fully captures holistic platform governance transparency or users' interaction with real-world governance UI/UX.

Data for Study 2 were collected over a three-week period from January 3, 2026 to January 24, 2026 using the same professional online recruitment channel as Study 1. Participants were eligible if they were at least 18 years old and had prior experience using digital platforms in everyday life. A total of 320 participants entered the experiment. After excluding respondents who failed the manipulation check, failed attention checks, or completed the experiment unrealistically quickly, 286 valid cases were retained. Among them, 144 were assigned to the high-transparency condition and 142 to the low-transparency condition.

Table 6 summarizes the Study 2 design. The full scenario materials used in the two transparency conditions are provided in Supplementary Appendix A.

**Table 6:** Design summary of Study 2.

Item	Description	Value
Design type	Between-subjects	Single factor, two conditions
Experimental factor	Transparency condition	High vs. low data governance transparency
Scenario platform	Platform type	Fictitious integrated platform (*LinkLife*)
Manipulation scope	Transparency cue	Structured vs. vague data-governance policy text
Data collection period	Fieldwork	January 3, 2026 to January 24, 2026
Recruitment source	Sample source	Professional online panel, mainland China
Initial sample	Participants entering experiment	320
Final sample	Valid participants	286
Group sizes	High/low transparency	144/142
Exclusion rules	Main criteria	Failed manipulation check, failed attention check, unrealistically fast completion

#### 3.3.3 Procedure

After providing informed consent, participants were randomly assigned by the survey system to one of the two transparency conditions. Randomization was implemented automatically at the questionnaire-platform level using a between-subjects allocation rule. Participants could not choose their condition, and the condition label was not shown to them. Each participant first read a short background introduction to the fictitious platform, followed by the condition-specific data-governance description.

To increase engagement and comprehension, the material was presented in a screenshot-like interface format resembling a platform data-policy summary page rather than a dense legal document. After reading the material, participants completed a brief comprehension item and a manipulation check measuring perceived transparency. They then responded to the main dependent measures, including trust, emotional regulation, and platform-related psychological wellbeing. To reduce carryover effects, the order of the three main scales was randomized across participants.

At the end of the experiment, respondents completed demographic questions and a short item measuring prior familiarity with similar platforms. The average experimental completion time was 7.84 min, and the median completion time was 7.21 min. Respondents who completed the study in less than 2.5 min were flagged for removal because such speed made meaningful reading of the scenario materials unlikely. The experimental procedure is summarized in Table 7.

**Table 7:** Procedure of Study 2.

Step	Task	Description
1	Consent	Participants read study information and provided consent
2	Random assignment	Participants assigned to high- or low-transparency condition
3	Scenario exposure	Participants read platform background and condition-specific material
4	Comprehension check	Brief item ensuring participants read core information
5	Manipulation check	Perceived transparency rated immediately after scenario
6	Main measures	Trust, emotional regulation, psychological wellbeing
7	Background questions	Demographics and platform familiarity
8	Data screening	Speeding, failed checks, missing data reviewed

#### 3.3.4 Manipulation check

The success of the transparency manipulation was assessed using a 3-item manipulation check scale rated on a 7-point Likert scale. The items focused on whether participants perceived the platform's data governance information as clear, understandable, and predictable. A successful manipulation would be indicated if participants in the high-transparency condition reported significantly higher perceived transparency than those in the low-transparency condition. In addition to the mean difference test, internal consistency of the manipulation check scale was examined to ensure that the manipulation captured a coherent perception rather than a single isolated reaction. This step was important because the experiment was designed to manipulate perceived transparency rather than mere exposure to a longer or shorter message.

The analysis of Study 2 proceeded in four steps. First, descriptive statistics were computed for each condition. Second, the manipulation check was conducted using an independent-samples *t*-test. Third, between-group differences in trust, emotional regulation, and platform-related psychological wellbeing were examined using independent-samples *t*-tests and one-way ANOVA, with effect sizes reported as Cohen's *d* or partial η^2^ where appropriate.

Fourth, conditional process analysis was used to examine whether the transparency manipulation was associated with psychological wellbeing indirectly through trust, emotional regulation, or the sequential pathway from trust to emotional regulation. Bootstrap confidence intervals based on 5,000 resamples were used to assess the stability of indirect effects (Hayes, 2022). This analysis strategy allowed the experiment to evaluate whether the manipulated transparency conditions reproduced the mechanism pattern suggested by the survey study.

## 4 Results

### 4.1 Results of study 1: survey test of the transparency–wellbeing mechanism

#### 4.1.1 Descriptive statistics and correlations

Before testing the proposed model, descriptive statistics and bivariate correlations were examined for all focal variables. As shown in Table 8, the focal variables were related in the theoretically expected directions. Perceived data governance transparency was positively correlated with trust, emotional regulation, and psychological wellbeing, thereby providing initial descriptive support for the proposed model. However, these bivariate associations should be interpreted as preliminary patterns rather than as evidence for the full explanatory structure, which was examined in the subsequent CFA, structural model, and mediation analyses.

**Table 8:** Descriptive statistics and correlations for Study 1 (*N* = 612).

Variable	Mean	SD	1	2	3	4	5	6
1. Perceived data governance transparency	4.91	1.03	1					
2. Trust	4.76	1.01	0.58^***^	1				
3. Emotional regulation	4.68	0.94	0.34^***^	0.47^***^	1			
4. Psychological wellbeing	4.83	0.97	0.41^***^	0.55^***^	0.52^***^	1		
5. Privacy concern	4.39	1.12	–0.19^***^	–0.23^***^	–0.14^**^	–0.21^***^	1	
6. Daily usage time	2.87	1.64	0.07	0.05	0.04	0.06	0.03	1

^***^p < 0.001, ^**^p < 0.01, ^*^p < 0.05.

**Figure 2:**
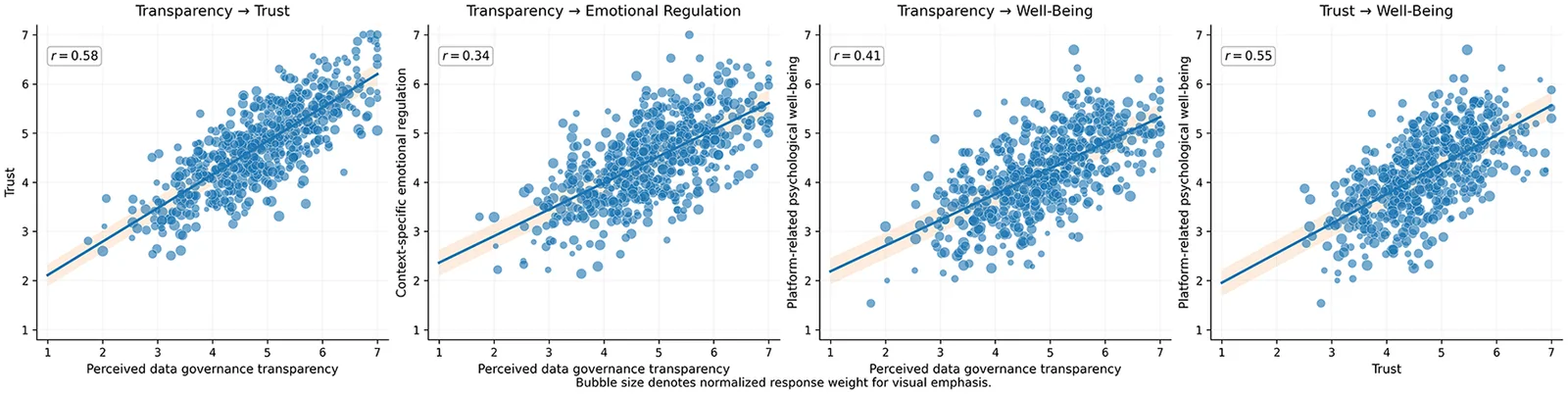
Bivariate relationship patterns among the core constructs in Study 1. Each panel visualizes the observed association between one focal predictor and one focal outcome.

The mean values indicate that respondents, on average, reported moderate levels of perceived transparency, trust, emotional regulation, and psychological wellbeing. Privacy concern showed small but significant negative correlations with the main focal variables, suggesting that users with stronger baseline privacy concerns tended to report lower trust and lower wellbeing in platform contexts. To complement the correlation matrix, Figure 2 visualizes the main bivariate relationship patterns among the focal constructs. The figure shows that the associations are not only statistically significant in the correlation table but also directionally consistent at the distributional level, with clearer perceived transparency tending to co-occur with higher trust, more stable emotional regulation, and better platform-related psychological wellbeing. Importantly, these plots are presented as descriptive visualizations of the observed association patterns rather than as substitutes for the structural model tested later.

**Figure 3:**
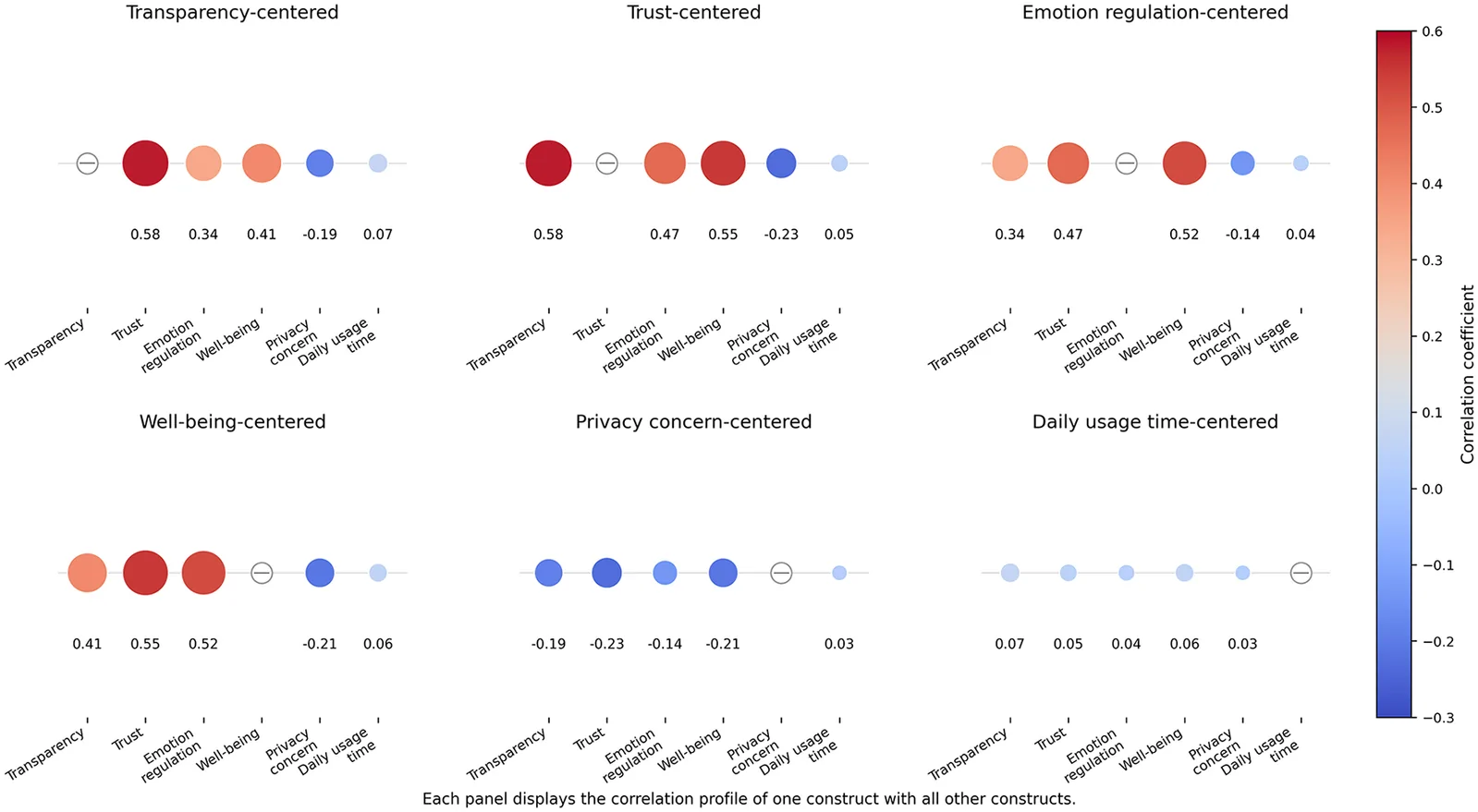
Construct-centered correlation profiles in Study 1. Each panel displays how one focal construct is related to all other constructs in the correlation matrix. Circle size reflects the absolute magnitude of the correlation coefficient, while color indicates its direction and strength.

Figure 3 provides a construct-centered view of the same correlation structure. Rather than emphasizing single pairwise relationships, this visualization highlights the broader pattern that transparency, trust, emotional regulation, and platform-related psychological wellbeing are positively clustered, whereas privacy concern shows negative associations with the main focal constructs and daily usage time remains only weakly related to them. This pattern is consistent with the interpretation that the proposed model is embedded in a coherent psychological structure rather than in isolated pairwise associations.

#### 4.1.2 Measurement model assessment

Confirmatory factor analysis was conducted to assess whether the four focal constructs—perceived data governance transparency, trust, emotional regulation, and psychological wellbeing—were empirically distinguishable. The hypothesized four-factor model showed acceptable fit to the data: χ^2^/*df* = 2.41, comparative fit index (CFI) = 0.953, Tucker–Lewis index (TLI) = 0.945, root mean square error of approximation (RMSEA) = 0.048, and standardized root mean square residual (SRMR) = 0.041. These indices were within commonly accepted thresholds, indicating that the proposed measurement model provided a reasonable representation of the data structure (Fornell and Larcker, 1981; Henseler et al., 2015). By comparison, alternative nested models combining conceptually distinct constructs showed substantially poorer fit. For example, a three-factor model combining trust and emotional regulation yielded CFI = 0.901 and RMSEA = 0.072, while a single-factor model produced very poor fit (CFI = 0.612, RMSEA = 0.139). These comparisons suggest that the focal constructs were not reducible to a single general evaluative factor. Reliability and validity statistics are reported in Table 9. Cronbach's alpha values ranged from 0.83 to 0.91, and composite reliability values ranged from 0.84 to 0.92, indicating satisfactory internal consistency. Standardized factor loadings were all above 0.65, and AVE values ranged from 0.52 to 0.66, supporting convergent validity. Discriminant validity was also acceptable, as the square roots of AVE exceeded the corresponding inter-construct correlations, and HTMT values remained below the recommended threshold. All focal constructs showed satisfactory internal consistency, and the convergent and discriminant validity evidence was within commonly accepted ranges (Fornell and Larcker, 1981; Henseler et al., 2015). Taken together, these results suggest that the measures were psychometrically adequate for subsequent structural analyses.

**Table 9:** Reliability and validity assessment for Study 1.

Construct	Cronbach's α	CR	AVE	Loading range
Perceived data governance transparency	0.89	0.90	0.60	0.69–0.82
Trust	0.91	0.92	0.66	0.74–0.86
Emotional regulation	0.83	0.84	0.52	0.65–0.77
Psychological wellbeing	0.88	0.89	0.57	0.68–0.81
Privacy concern	0.84	0.85	0.59	0.67–0.79

#### 4.1.3 Model testing

Structural equation modeling was used to examine the proposed explanatory model while controlling for age, gender, education, platform use frequency, daily usage time, platform type, and privacy concern. The structural model demonstrated acceptable fit to the data: χ^2^/*df* = 2.56, CFI = 0.947, TLI = 0.939, RMSEA = 0.051, and SRMR = 0.046. The results showed that perceived data governance transparency was positively associated with psychological wellbeing (β = 0.17, *p* < 0.01), trust (β = 0.59, *p* < 0.001), and emotional regulation (β = 0.16, *p* < 0.01). Trust was positively associated with emotional regulation (β = 0.38, *p* < 0.001) and psychological wellbeing (β = 0.29, *p* < 0.001). Emotional regulation was positively associated with psychological wellbeing (β = 0.34, *p* < 0.001). These results indicate that the proposed pattern of relationships was broadly supported in the survey data.

As shown in Table 10, all hypothesized structural paths were significant and in the expected directions. The magnitude and direction of these coefficients suggest that the proposed model captured a coherent set of relationships among perceived transparency, trust, emotional regulation, and psychological wellbeing.

**Table 10:** Structural path estimates for Study 1.

Path	Standardized coefficient (β)	Result
Transparency → psychological wellbeing	0.17^**^	Supported
Transparency → trust	0.59^***^	Supported
Transparency → emotional regulation	0.16^**^	Supported
Trust → psychological wellbeing	0.29^***^	Supported
Trust → emotional regulation	0.38^***^	Supported
Emotional regulation → psychological wellbeing	0.34^***^	Supported

^***^p < 0.001, ^**^p < 0.01, ^*^p < 0.05.

Taken together, these findings provide initial support for H1 and for the broader structure of the proposed model. At the same time, the persistence of a significant direct association between transparency and wellbeing suggests that the indirect mechanisms may be partial rather than fully exhaustive.

#### 4.1.4 Mediation and sequential mediation analysis

To examine the indirect pathways specified in H2, H3, and H4, bias-corrected bootstrap mediation analysis with 5,000 resamples was conducted (Hayes, 2022). As shown in Table 11, none of the bootstrap confidence intervals included zero, indicating that the indirect effects were statistically reliable. This pattern supports the interpretation that trust and emotional regulation functioned not only as separate explanatory pathways but also as an ordered sequential mechanism. First, trust significantly mediated the relationship between perceived data governance transparency and psychological wellbeing (indirect effect = 0.171, 95% CI [0.116, 0.236]). Second, emotional regulation also significantly mediated the relationship (indirect effect = 0.054, 95% CI [0.022, 0.096]). Third, the sequential indirect effect of transparency on psychological wellbeing through trust and emotional regulation was significant (indirect effect = 0.077, 95% CI [0.049, 0.113]). Since none of the confidence intervals included zero, the indirect effects were statistically supported. These findings suggest that trust and emotional regulation each functioned as meaningful explanatory pathways and, more importantly, that the data were consistent with a sequential mechanism in which transparency was associated with greater trust, greater trust was associated with more stable emotional regulation, and emotional regulation was associated with higher psychological wellbeing.

**Table 11:** Bootstrap mediation results for Study 1.

Indirect path	Indirect effect	95% CI	Interpretation
Transparency → trust → wellbeing	0.171	[0.116, 0.236]	Significant
Transparency → emotional regulation → wellbeing	0.054	[0.022, 0.096]	Significant
Transparency → trust → emotional regulation → wellbeing	0.077	[0.049, 0.113]	Significant

Overall, the mediation results provide support for H2, H3, and H4. Importantly, the sequential mechanism was not simply a statistical add-on but represented a non-trivial portion of the total indirect effect, suggesting that governance-related perceptions may be linked to wellbeing through an organized psychological process rather than only through isolated one-step associations.

#### 4.1.5 Robustness analyses before boundary-condition testing

Several supplementary analyses were conducted to assess the stability of the Study 1 results before the formal moderation test. First, multi-group analyses across primary platform type (social, e-commerce, mobility, and life-service platforms) indicated that the general pattern of the model remained stable, although the path from transparency to trust was somewhat stronger among e-commerce and life-service users than among mobility users. Second, the model was re-estimated after excluding respondents with extremely high daily usage time to ensure that the results were not driven by heavy users alone. The main coefficients remained substantively similar.

These analyses were treated as robustness checks rather than as substitutes for the theoretically specified boundary-condition test. Privacy concern was not relegated to a *post-hoc* median split. Instead, it was tested as the moderator specified in H5 through an interaction model, with simple-slope analysis reported in the dedicated boundary-condition section below.

#### 4.1.6 Alternative model comparison

To evaluate whether the proposed sequential mechanism represented the most plausible explanatory structure, additional model comparisons were conducted. In particular, the hypothesized sequential model was compared with two theoretically relevant alternatives: (1) a parallel mediation model in which trust and emotional regulation simultaneously mediated the relationship between perceived data governance transparency and psychological wellbeing without a directional path between the two mediators, and (2) a reversed sequential model in which emotional regulation preceded trust. As shown in Table 12, the proposed sequential model demonstrated the best overall fit among the three specifications. Although the parallel mediation model also showed acceptable fit, it did not capture the theoretically expected directional linkage between trust and emotional regulation. By contrast, the reversed sequential model yielded comparatively poorer fit. These comparisons suggest that the trust-to-emotional-regulation ordering provides a more plausible representation of the mechanism pattern observed in this study.

**Table 12:** Comparison of the proposed and alternative models.

Model	Structure	χ^2^/*df*	CFI	TLI	RMSEA
Model 1	Proposed sequential model: transparency → trust → emotional regulation → wellbeing	2.56	0.947	0.939	0.051
Model 2	Parallel mediation model: transparency → trust/emotional regulation → wellbeing	2.71	0.941	0.932	0.054
Model 3	Reversed sequential model: transparency → emotional regulation → trust → wellbeing	2.94	0.934	0.924	0.058

For Study 1, the proposed sequential model showed better overall fit than the alternative specifications. The hypothesized model yielded acceptable fit indices (χ^2^/*df* = 2.56, CFI = 0.947, TLI = 0.939, RMSEA = 0.051, SRMR = 0.046). The parallel mediation model also showed acceptable fit, but the overall explanatory structure was weaker, and the sequential association between trust and emotional regulation was not captured. By contrast, the reversed sequential model demonstrated comparatively poorer fit and weaker indirect effects. The comparison suggests that trust and emotional regulation are not best understood as merely parallel pathways. Rather, the data were more consistent with a process in which perceived transparency first shaped users' trust in the platform and then influenced the stability of their emotional regulation in relation to data-related uncertainty. This ordering is theoretically consistent with the view that confidence in the platform reduces vigilance and psychological threat, thereby facilitating more stable emotion regulation (Mayer et al., 1995; Gross, 1998).

A similar comparison was conducted in Study 2. The experimental data again showed that the proposed sequence from trust to emotional regulation provided a more coherent account of the observed condition differences than either a purely parallel mediation structure or the reversed ordering. Taken together, these results strengthen confidence that the proposed sequential model is not simply one of many equally plausible specifications, but a theoretically and empirically preferable representation of the mechanism pattern observed in this study.

#### 4.1.7 Common method variance and multicollinearity diagnostics

Additional diagnostic analyses were conducted to address common method variance and multicollinearity concerns. Harman's single-factor test was used only as a preliminary check. The first unrotated factor accounted for 31.84% of the total variance, which was below the level that would suggest dominance by a single method factor. More importantly, the single-factor CFA model showed substantially poorer fit than the hypothesized multi-factor measurement model (CFI = 0.612, TLI = 0.571, RMSEA = 0.139, SRMR = 0.118), indicating that the observed covariance structure was not adequately explained by a single common factor.

To provide a more rigorous assessment, a latent method factor model was estimated. After adding a common latent method factor, the main substantive paths remained stable in direction and significance. Specifically, the paths from perceived data governance transparency to trust (β = 0.56, *p* < 0.001), from trust to emotional regulation (β = 0.36, *p* < 0.001), and from emotional regulation to platform-related psychological wellbeing (β = 0.32, *p* < 0.001) remained significant. The sequential indirect effect also remained statistically supported (indirect effect = 0.069, 95% CI [0.043, 0.103]). A marker-variable specification yielded the same substantive conclusion. These results suggest that the proposed mechanism was not primarily driven by common method variance.

Multicollinearity was assessed using variance inflation factors. The VIF values for the focal predictors ranged from 1.02 to 1.87, all below commonly used thresholds for problematic multicollinearity. Therefore, the structural estimates were unlikely to be distorted by excessive overlap among predictors. The detailed diagnostic results are summarized in Table 13.

**Table 13:** Common method variance and multicollinearity diagnostics.

Diagnostic area	Test or predictor	Result	Interpretation
Common method variance	Harman's single-factor test	First factor = 31.84%	No single dominant factor observed
Common method variance	Single-factor CFA	CFI = 0.612; RMSEA = 0.139	Poorer fit than the hypothesized model
Common method variance	Latent method factor model	Key paths remained significant	Findings stable after accounting for shared method variance
Common method variance	Marker-variable specification	Key paths remained significant	Findings not primarily method-driven
Multicollinearity	Perceived data governance transparency	VIF = 1.64	Acceptable
Multicollinearity	Trust	VIF = 1.87	Acceptable
Multicollinearity	Emotional regulation	VIF = 1.42	Acceptable
Multicollinearity	Privacy concern	VIF = 1.08	Acceptable
Multicollinearity	Daily usage time	VIF = 1.02	Acceptable

#### 4.1.8 Privacy concern as a boundary condition

To examine H5, privacy concern was tested as a continuous moderating variable rather than by relying on a median split. Perceived data governance transparency and privacy concern were mean-centered, and their interaction term was added to the model predicting platform-related psychological wellbeing. The interaction between perceived transparency and privacy concern was significant (β = −0.08, *p* = 0.018), indicating that the association between perceived transparency and platform-related psychological wellbeing differed depending on users' baseline privacy concern.

Simple-slope analysis further clarified the pattern. Among users with lower privacy concern, perceived data governance transparency was more strongly associated with platform-related psychological wellbeing (β = 0.24, *p* < 0.001). Among users with higher privacy concern, the association was weaker but remained significant (β = 0.10, *p* = 0.042). This pattern is consistent with H5 and suggests that transparency may yield stronger psychological benefits when users are less strongly predisposed to interpret platform data practices through a risk-sensitive lens. For users with high privacy concern, transparent communication may still support understanding and trust, but its direct psychological comfort effect appears more limited because clearer data-governance information can also heighten awareness of data exposure and platform discretion.

These results support the boundary-condition argument developed in the theory section. The findings do not imply that privacy-concerned users reject transparency. Rather, they suggest that transparency has a more complex psychological meaning for these users: it may improve understanding while simultaneously making data risks more salient. Table 14 reports the moderation results.

**Table 14:** Moderating effect of privacy concern in Study 1.

Effect	Standardized coefficient	*p* value	Interpretation
Transparency → wellbeing	0.17	0.006	Positive direct association
Privacy concern → wellbeing	–0.13	0.011	Higher concern associated with lower wellbeing
Transparency × privacy concern → wellbeing	–0.08	0.018	Privacy concern weakens the transparency–wellbeing link
Simple slope: low privacy concern	0.24	<0.001	Stronger positive association
Simple slope: high privacy concern	0.10	0.042	Weaker positive association

### 4.2 Results of study 2: scenario-based experimental validation

A manipulation check was conducted to verify whether the two experimental conditions produced distinct perceptions of data governance transparency. As expected, participants in the high-transparency condition reported significantly higher perceived transparency (*M* = 5.42, SD = 0.88) than those in the low-transparency condition (*M* = 3.67, SD = 1.01), *t*_(284)_ = 15.86, *p* < 0.001, Cohen's *d* = 1.88. This result indicates that the scenario manipulation successfully altered participants' subjective perception of platform transparency.

#### 4.2.1 Group comparisons

Following the successful manipulation check, group differences in trust, emotional regulation, and psychological wellbeing were examined. As shown in Table 15, participants in the high-transparency condition scored higher on all three focal variables than those in the low-transparency condition.

**Table 15:** Group comparison results for Study 2.

Variable	High transparency (*n* = 144)	Low transparency (*n*=142)	*t*	*p*	Cohen's *d*
Perceived transparency	5.42 (0.88)	3.67 (1.01)	15.86	<0.001	1.88
Trust	5.01 (0.96)	4.18 (1.03)	7.07	<0.001	0.84
Emotional regulation	4.86 (0.89)	4.39 (0.94)	4.34	<0.001	0.51
Psychological wellbeing	5.02 (0.91)	4.41 (0.99)	5.44	<0.001	0.64

Entries for group means are reported as mean (standard deviation).

More specifically, trust was significantly higher in the high-transparency condition (*M* = 5.01, SD = 0.96) than in the low-transparency condition (*M* = 4.18, SD = 1.03), *t*(284) = 7.07, *p* < 0.001, Cohen's *d* = 0.84. Context-specific emotional regulation was also higher in the high-transparency condition (*M* = 4.86, SD = 0.89) than in the low-transparency condition (*M* = 4.39, SD = 0.94), *t*_(284)_ = 4.34, *p* < 0.001, Cohen's *d* = 0.51. Platform-related psychological wellbeing showed a similar pattern, with participants in the high-transparency condition reporting higher wellbeing (*M* = 5.02, SD = 0.91) than those in the low-transparency condition (*M* = 4.41, SD = 0.99), *t*_(284)_ = 5.44, *p* < 0.001, Cohen's *d* = 0.64.

**Figure 4:**
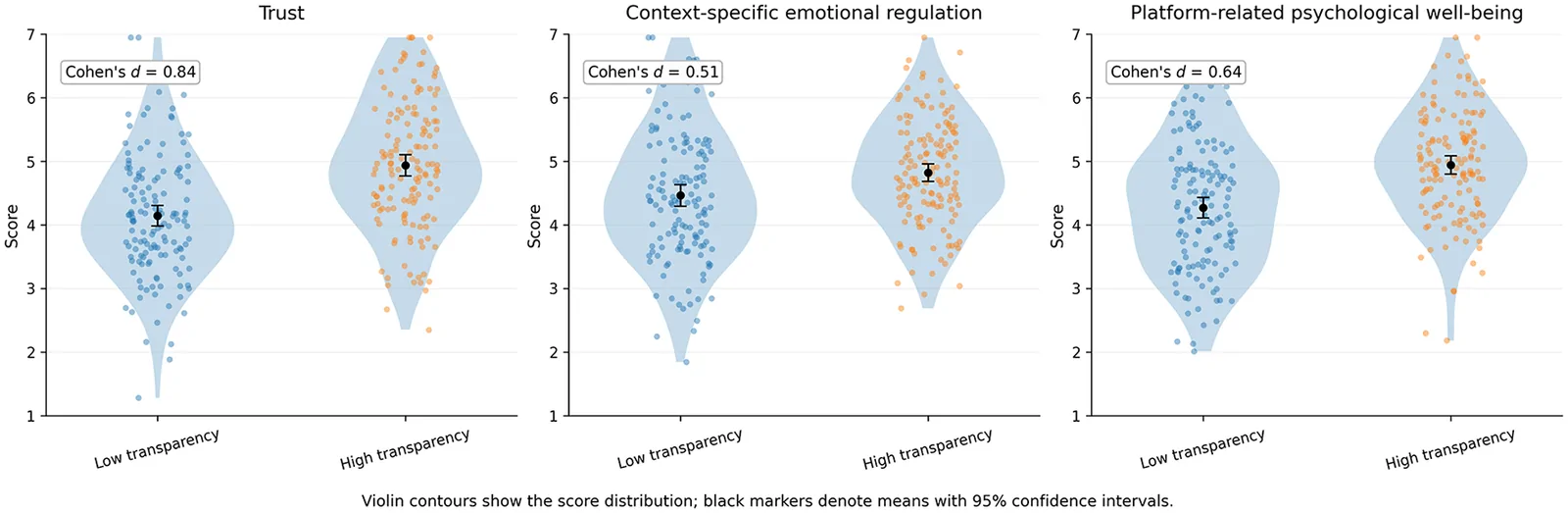
Distributional differences across transparency conditions in Study 2. The violin contours show the distribution of scores in the low-transparency and high-transparency conditions, and the black markers denote group means with 95% confidence intervals.

Figure 4 complements the mean comparisons in Table 15 by showing the full score distributions under the two transparency conditions. In addition to the separation of group means, the figure indicates an overall upward shift in the distributions of trust, context-specific emotional regulation, and platform-related psychological wellbeing in the high-transparency condition. This pattern suggests that the observed condition differences were not driven merely by a small number of extreme observations, but reflected a broader shift in participants' psychological responses. Taken together, these results indicate that experimentally induced differences in transparency conditions were associated with systematic differences in the focal psychological outcomes.

#### 4.2.2 Mechanism-oriented analysis

To examine whether the experimental condition reproduced the mechanism pattern observed in Study 1, a conditional process analysis with 5,000 bootstrap resamples was conducted (Hayes, 2022). The transparency condition (high = 1, low = 0) was entered as the independent variable, psychological wellbeing as the dependent variable, and trust and emotional regulation as sequential mediators. The indirect effect of transparency condition on psychological wellbeing through trust was significant (indirect effect = 0.214, 95% CI [0.131, 0.311]). The indirect effect through emotional regulation alone was smaller but still significant (indirect effect = 0.061, 95% CI [0.018, 0.118]). Most importantly, the sequential indirect effect through trust and emotional regulation was significant (indirect effect = 0.072, 95% CI [0.036, 0.122]). After the mediators were included, the direct effect of transparency condition on psychological wellbeing remained significant but was reduced in magnitude, indicating partial mediation. These experimental results are consistent with the pattern observed in Study 1. Although the two studies rely on different forms of evidence, both suggest that transparency-related differences are linked to wellbeing not only directly, but also through trust and emotional regulation, including a sequential path from trust to emotional regulation.

### 4.3 Summary of results across the two studies

Across both studies, the findings were generally consistent with the proposed explanatory model. In the survey study, perceived data governance transparency was positively associated with platform-related psychological wellbeing, and both trust and emotional regulation functioned as significant mediators. The additional diagnostics indicated that the core findings were not primarily attributable to common method variance or multicollinearity. The moderation analysis further showed that privacy concern weakened the direct transparency–wellbeing association, suggesting that transparency does not yield identical psychological benefits for all users.

In the scenario experiment, manipulated transparency conditions produced systematic differences in trust, emotional regulation, and platform-related psychological wellbeing, and the same indirect mechanism pattern was observed. Taken together, the results suggest that perceived data governance transparency may be psychologically consequential in digital platform environments. More importantly, the findings indicate that its relevance to wellbeing is not limited to a simple direct association. Rather, the evidence supports the interpretation that governance-related perceptions may become linked to users' wellbeing through an organized psychological process involving trust and emotional regulation, while privacy concern defines an important boundary condition.

## 5 Discussion

### 5.1 Summary of findings

The present research set out to examine whether and how perceived data governance transparency in digital platforms may be associated with users' platform-related psychological wellbeing. Rather than assuming that transparency is inherently beneficial, the study proposed an explanatory model in which trust and emotional regulation were positioned as two key psychological mechanisms. Across both the survey study and the scenario experiment, the findings were broadly consistent with this model.

First, perceived data governance transparency was positively related to platform-related psychological wellbeing. Users who perceived platform data governance practices as clearer, more understandable, and more predictable tended to report more positive psychological experiences, including greater comfort, safety, and subjective ease in platform use. Second, trust and emotional regulation each mediated this relationship, and the sequential pathway from transparency to trust, from trust to emotional regulation, and from emotional regulation to wellbeing was supported. Third, privacy concern moderated the direct transparency–wellbeing association. The positive association was weaker among users with higher privacy concern, suggesting that transparency can be psychologically beneficial but not uniformly so.

### 5.2 Theoretical implications

This study offers several theoretical implications. First, it refines the conceptualization of perceived data governance transparency in digital platforms. Rather than equating transparency with information clarity, privacy disclosure, perceived control, algorithmic transparency, or trust, the study conceptualizes it as a user-perceived governance cue concerning the understandability, predictability, and accountability of platform data practices. This distinction helps address potential redundancy with adjacent constructs and clarifies why transparency can be psychologically meaningful even when it is not equivalent to actual user control.

Second, the study extends platform governance research from behavioral and institutional outcomes to platform-related psychological wellbeing. Existing research has often emphasized whether transparency improves compliance, adoption, trust, or continued use. The present study shows that transparency may also be associated with users' subjective psychological experience within the platform environment. This shift is theoretically important because digital platforms are not only service systems but also data-governed environments in which users interpret uncertainty, vulnerability, and institutional responsibility.

Third, the study contributes to trust theory by positioning trust as part of a broader psychological sequence rather than merely as a terminal outcome of transparency. The findings suggest that trust may reduce users' need for vigilance and perceived vulnerability, thereby facilitating more stable emotional regulation. This mechanism helps explain how governance perceptions may be translated into wellbeing through an organized psychological process.

Fourth, the study advances a context-specific account of emotional regulation in platform governance. Emotional regulation is theorized and measured as users' perceived ability to manage unease and emotional instability arising from data-related uncertainty. This approach extends emotion regulation theory into a digital governance context and explains why the emotional consequences of platform transparency should not be treated as peripheral. The results indicate that users' capacity to regulate data-related unease is a meaningful mechanism connecting governance perceptions with platform-related psychological wellbeing.

Fifth, by introducing privacy concern as a formal boundary condition, the study avoids a universalistic account of transparency. The moderation analysis suggests that transparency may not provide the same psychological benefit for all users. Users with stronger privacy concern may process transparency cues more cautiously because clearer information can simultaneously reduce ambiguity and heighten awareness of data-related risks. This boundary-aware interpretation strengthens the theoretical precision of the model and clarifies for whom transparency is more likely to produce psychological dividends.

Finally, the study clarifies its incremental contribution relative to prior transparency–trust research. The novelty of the model does not lie in claiming that transparency can influence trust, which is already well established. Rather, the contribution lies in explaining how trust becomes psychologically consequential through context-specific emotional regulation and in showing that this process is conditioned by privacy concern. In this sense, the study moves from a simple transparency–trust–outcome association to a mechanism- and boundary-aware account of platform-related psychological wellbeing.

### 5.3 Practical implications

The findings also have several practical implications for platform operators and policymakers. For platform firms, the results suggest that data governance transparency should not be treated solely as a compliance requirement or reputational signal. It may also function as a psychological design feature that shapes how users experience the platform. If data-related rules, disclosures, and explanations are perceived as vague or difficult to interpret, users may not only distrust the platform but also experience lower psychological comfort and greater unease. By contrast, governance communication that is clearer, more structured, and more predictable may help create a more psychologically supportive platform environment (Esmaeilzadeh, 2019; Peters et al., 2018; Paunov et al., 2022).

This has implications for the design of privacy notices, data consent flows, in-app policy explanations, and user-facing governance interfaces. Firms should pay attention not only to legal completeness but also to perceived intelligibility. From a user-experience perspective, effective governance transparency should make data practices understandable without overwhelming users with technical or legal complexity. Transparent governance communication may therefore be most useful when it is concise, layered, context-sensitive, and connected to concrete user rights and actions. The findings also suggest that trust-building and emotional reassurance should be considered part of responsible platform governance. When designing data governance communication, firms may benefit from considering how users are likely to interpret and emotionally respond to the information presented. This means that governance design should not be reduced to formal disclosure volume. It should also consider whether users can understand what is being communicated, whether the explanation reduces ambiguity, and whether the interface supports a sense of psychological stability rather than merely satisfying formal requirements.

For policymakers and regulators, the study suggests that transparency standards may need to go beyond the formal disclosure of information. Regulations that require platforms to disclose data practices are important, but the present findings imply that disclosure may be most meaningful when users can actually perceive it as clear, comprehensible, and predictable. In this sense, user-centered intelligibility may be as important as formal transparency (Cheong, 2024; Moon et al., 2025; Shin, 2025). Governance frameworks that incorporate readability, interpretability, and user-perceived clarity may be better aligned with the psychological realities of digital platform use.

### 5.4 Boundary considerations

Although the findings support the proposed explanatory model, they should not be interpreted as showing that transparency is universally and unconditionally beneficial in every digital context. The present study suggests that, under the conditions examined here, higher perceived data governance transparency was associated with more positive psychological responses. However, this does not imply that greater transparency will always improve wellbeing regardless of content, context, user characteristics, or institutional environment.

First, privacy concern represents an important boundary condition. For users with lower privacy concern, transparent governance communication may primarily function as a signal of responsibility, predictability, and user-centered design. For users with higher privacy concern, the same transparency cues may also make the scope and complexity of data practices more salient. This helps explain why the direct psychological benefit of transparency may be weaker among highly privacy-concerned users.

Second, algorithmic literacy may represent another important but untested boundary condition. Users with higher algorithmic literacy may process data-governance information differently because they can better infer how data collection, profiling, recommendation, and personalization practices are connected. In some cases, such literacy may make transparency more empowering; in other cases, it may intensify awareness of data dependence. Because algorithmic literacy was not measured in the present design, the study does not draw empirical conclusions about this moderator. Future research should examine whether algorithmic literacy strengthens, weakens, or reshapes the psychological effects of transparency.

Third, platform type may matter. Transparency in a social media platform, where visibility, social comparison, and interpersonal exposure are salient, may operate differently from transparency in e-commerce, mobility, or life-service platforms, where transaction security, location data, and service reliability may be more central. Therefore, the psychological meaning of transparency may depend on the type of data risk that users associate with a particular platform category.

Fourth, cultural and institutional context may shape how users interpret data governance transparency. The present study was conducted with adult digital platform users recruited through online panels in mainland China. Users in other regulatory environments may hold different expectations about platform accountability, privacy rights, institutional protection, and the credibility of platform disclosures. In contexts with stronger regulatory trust, transparency may reinforce confidence. In contexts with lower institutional trust or stronger privacy skepticism, transparency may be interpreted more cautiously. Thus, the findings should be generalized across cultures and regulatory systems with care.

Fifth, the experimental evidence should be interpreted within the scope of the manipulation. Study 2 used a fictitious platform and manipulated transparency through structured vs. vague data-governance text. This design improved internal control and reduced prior brand bias, but it primarily captured responses to a policy-text-readability and policy-structure cue. Real-world transparency is communicated through broader governance interactions, including interface design, consent mechanisms, privacy dashboards, algorithmic explanations, notification systems, and support channels. Therefore, the experimental results should not be overstated as direct evidence about fully implemented platform governance systems or holistic UI/UX-based transparency.

### 5.5 Limitations

Several limitations should be acknowledged. First, although the study combined survey and experimental methods, much of the evidence still relied on self-reported psychological measures. Self-report is appropriate for constructs such as trust, emotional regulation, and platform-related psychological wellbeing, but it may also be influenced by response styles, social desirability, or contextual framing. To address this concern, the revised analysis included additional common method variance diagnostics, including single-factor CFA comparison, latent method factor analysis, and marker-variable analysis. Nevertheless, future research could complement self-report data with behavioral indicators, experience-sampling designs, interface-use logs, or physiological measures of stress and emotional response.

Second, Study 1 was cross-sectional. Although the hypothesized ordering was theoretically grounded and supported by alternative model comparisons, cross-sectional data cannot fully establish temporal sequence. The scenario experiment in Study 2 provided complementary evidence by manipulating transparency conditions, but longitudinal studies are still needed to examine how transparency perceptions, trust, emotional regulation, and wellbeing evolve over time.

Third, Study 2 used a fictitious platform and a text-based transparency manipulation. This approach helped control for prior platform attitudes and isolate the effect of transparency-related information, but it also reduced ecological richness. The manipulation mainly tested users' responses to differently structured data-governance descriptions. It therefore captured policy-text readability, specificity, and structure rather than the full experience of interacting with real platform governance systems. Future studies should examine real-world interfaces, interactive consent flows, privacy dashboards, layered notices, notification cues, support channels, and field experiments to determine whether the mechanism observed here generalizes to more naturalistic settings.

Fourth, the sample was drawn from adult digital platform users recruited through online panels in mainland China. This sampling frame was appropriate for the present research context, but it limits generalizability across cultural and regulatory environments. Users in other countries may interpret transparency differently depending on privacy norms, institutional trust, platform regulation, and prior experience with data protection. Cross-cultural and cross-institutional comparisons would therefore be valuable.

Finally, the present model focused on trust, emotional regulation, and privacy concern. Although these constructs were theoretically justified, they do not exhaust all possible mechanisms and boundary conditions. Other factors such as perceived control, cognitive load, perceived fairness, algorithmic literacy, prior privacy harm, platform dependence, and prior negative experience with data misuse may also influence how transparency becomes psychologically consequential. Future research could extend the model by examining these additional explanatory pathways.

## 6 Conclusion

This study proposed and examined an explanatory model linking perceived data governance transparency in digital platforms to users' platform-related psychological wellbeing through trust and context-specific emotional regulation. Across a survey study and a scenario experiment, the findings were broadly consistent with the view that perceived transparency is psychologically consequential in platform environments. Users who perceived greater transparency tended to report higher platform-related psychological wellbeing, and this relationship was partially explained by stronger trust and more stable emotional regulation.

More importantly, the findings suggest that the relevance of data governance transparency to wellbeing involves an organized psychological process rather than a simple one-step association. Trust and emotional regulation did not merely coexist as separate correlates; they formed a meaningful sequence through which governance perceptions may become translated into subjective platform experience. At the same time, privacy concern weakened the direct transparency–wellbeing association, indicating that transparency is more psychologically beneficial under some user conditions than others.

Overall, the present research suggests that data governance transparency is not only an institutional or regulatory issue, but also a psychologically relevant environmental feature. The value of transparency depends on whether users perceive data-governance arrangements as understandable, predictable, and accountable, and on whether these perceptions are converted into trust, emotional stability, and platform-related psychological comfort. The results also caution against overgeneralization: the experimental evidence reflects responses to structured vs. vague policy text, and future work should test richer forms of interactive, UI-based, and field-level governance transparency.

## Data Availability

The raw data supporting the conclusions of this article will be made available by the authors, without undue reservation.
